# Electrocardiographic sex index: a continuous representation of sex

**DOI:** 10.1186/s13293-025-00727-2

**Published:** 2025-07-17

**Authors:** Ibrahim Karabayir, Turgay Celik, Luke Patterson, Liam Butler, David Herrington, Oguz Akbilgic

**Affiliations:** Department of Cardiovascular Medicine, Wake Forest School of Medicine, Winston- Salem, NC 27157 USA

**Keywords:** Sex, Electrocardiogram, Artificial intelligence, Clinical risk prediction, Heart failure

## Abstract

Clinical risk calculators consider sex as a binary variable. However, sex is a complex trait with anatomic, physiologic, and metabolic attributes that are not easily summarized in this manner [1]. We propose a continuous representation of sex, the ECG Sex Index (ESI), derived via artificial intelligence analyses of electrocardiograms (ECG-AI).

We used an ECG repository at Wake Forest Baptist Health (Winston-Salem, NC) to develop a convolutional neural network-based ECG-AI model to detect sex from standard 12-lead ECGs. We utilized a rank-ordered transformation of the outcomes of ECG-AI to create the ESI. We also created a sex discordance index (SDI) from the ESI and assessed its utility in 1-year risk prediction for all-cause mortality, heart failure, and kidney failure.

The Wake Forest cohort included 3,573,844 ECGs and electronic health record data from 754,761 patients; 75% were White, 17% were Black, and 51% were female, with a mean age (SD) of 61 (17) years. The PhysioNet external validation cohort included 45,152 ECGs from 10,646 patients from two hospitals in China. The PhysioNet cohort was 100% Asian, 43.6% female, and had a mean age (SD) of 59 (20) years. ECG-AI provided a holdout area under the curve of 0.95 and an external validation area under the curve of 0.92. Lower ESI scores in males and higher ESI scores in females were associated with a greater risk for clinical outcomes. The ESI and SDI demonstrated comparable accuracy to binary sex in logistic regression analyses and outperformed binary sex in predicting clinical outcomes, highlighting their value as predictors in risk calculators for all-cause mortality, heart failure, and kidney failure.

**Supplementary Information:**

The online version contains supplementary material available at 10.1186/s13293-025-00727-2.

## Abstract (plain language summary)

An individual’s sex is typically classified as male or female at birth based on the presence of either a penis or a clitoris. However, there are many common features of male and female individuals at the metabolic and cellular levels. These commonalities are neglected in disease risk prediction models when an individual is labeled as male or female. This study proposes a novel, nonbinary representation of sex to be used in disease risk prediction models. Our novel nonbinary variable is derived via artificial intelligence applied to electrocardiographic data. It predicts all-cause mortality, heart failure, and kidney failure better than a binary sex variable.

**Supplementary Information:**

The online version contains supplementary material available at 10.1186/s13293-025-00727-2.

## Highlights

We introduce an ECG-AI-driven continuous representation of sex, the ESI, which is a better predictor of cardiovascular and non-cardiovascular disease than binary sex.

The difference between an individual’s binary sex designation and their ESI value is associated with an increased risk for all-cause mortality, heart failure, and kidney failure.

A continuous representation of sex, the ESI, should be considered in risk prediction models for cardiovascular and renal outcomes, including mortality, heart failure, and kidney failure, where sex is hypothesized to be a relevant factor.

**Supplementary Information:**

The online version contains supplementary material available at 10.1186/s13293-025-00727-2.

## Introduction

At the molecular level, the expression of Y chromosome genes and their interactions with genes from the rest of the genome are far from binary, resulting in powerful, but typically graded, effects on an extraordinary array of downstream anatomic, physiologic, and metabolic traits [2]. Unfortunately, in most clinical and epidemiologic research, as well as in clinical risk prediction models, sex has a binary variable to represent sex-specific effects on traits not otherwise directly measured [1]. This approach presupposes that sex-specific variation in unmeasured attributes can be adequately summarized in a binary fashion. Our goal was to develop a continuous metric to represent sex-specific effects on human biological attributes, moving beyond a binary approach. This continuous measure could enhance clinical research and care, particularly in predicting risk for various clinical outcomes. We hypothesized that a continuous measure of sex effects on clinically relevant, yet unmeasured traits could improve research and risk prediction for outcomes such as all-cause mortality, heart failure, and kidney failure.

We derived a novel measure via state-of-the-art artificial intelligence (AI) techniques applied to data from digital 12-lead electrocardiograms (ECG). ECGs encode complex information about cardiac structure and function, often revealing binary differences in sex, and importantly, provide insights into clinical and physiologic traits—such as heart rate variability and QT interval duration—that also vary significantly by sex [3, 4]. Moreover, and critically important to the current work, ECG data encode information about a variety of clinical and physiologic traits, many of which also exhibit significant variation by sex. Application of AI to ECG data (ECG-AI) has demonstrated that information about many of these traits can be retrieved from a suitably designed deep learning model that includes but is not limited to cardiovascular diseases [5–9], arrhythmias [10–13], neurodegenerative diseases [14, 15], hyperkalemia [16], and all-cause mortality [17]. With this in mind, we hypothesized that ECG-AI could produce a simple, inexpensive, and integrated measure of how an individual’s sex chromosomal composition affects clinical and physiologic attributes that are relevant for human health.

To test this hypothesis, we developed and validated an ECG-AI model producing a continuous metric of sex effects based on ECGs from a large cohort of racially, demographically, and geographically diverse adults. We then compared the informativeness of this novel measure with a conventional binary representation of sex in several well-established cardiovascular and non-cardiovascular risk prediction models, focused on the outcomes of all-cause mortality, heart failure, and kidney failure. If successful, this approach could have implications for a wide array of clinical decision-making as well as basic, clinical and population research where accounting for sex effects is important. To facilitate reproducibility and widespread use of our AI models, the sex classification and ESI calculation code will be freely available for the research community via the GitHub repository.

## Methods

### Data sources

This project used retrospective data from the Wake Forest University School of Medicine (Winston-Salem, NC), and was approved by its Institutional Review Board. This ECG repository includes 3,573,844 12-lead, 10-second resting ECGs linked to the electronic health records (EHR) data of 754,761 patients. In this cohort, 75% were White, 17% were Black, and 51% were female, with a mean age (SD) of 61 (17) years. All ECGs were collected as part of routine clinical care, independent of patient health status, reflecting any patient who had an ECG collected. Acknowledging that ECG is typically ordered for clinical reasons, the cohort represent a slightly biased cohort of people who needed ECG, which was reflected on the distribution of clinical conditions within the cohort with 7.5% heart failure, 10.0% coronary artery disease, 8.0% chronic kidney disease, 6.7% valvular disease, 7.3% arrhythmias, 13.1% diabetes, and 26.4% hypertension rate, while 65.7% of the analytical cohort being free of any of these conditions.

We also used the publicly available dataset from PhysioNet entitled “A large-scale 12-lead electrocardiogram database for arrhythmia study” for external validation of the ECG-AI models [18–20]. The PhysioNet dataset contains 45,152 12-lead, 10-second ECGs from 10,646 patients at Shaoxing People’s Hospital, China, and Ningbo First Hospital, China. In this dataset, 100% of patients were Asian and 43.6% were female, with a mean age (SD) of 59.2 (19.5) years.

### ECG data Preparation

Wake Forest ECGs were stored in the GE MUSE system and exported as XML files, including base 64-encoded waveform data. We developed a customized Python library to parse and decode 12-lead waveform ECG data into individual NumPy files, and the associated metadata were stored in CSV files. The ECGs sampled at 500 Hz were down sampled to 250 Hz. Hence, each ECG was represented as a 2500 × 12 matrix, with rows representing time in milliseconds and columns representing voltage from each lead in microvolts. The PhysioNet ECGs were similarly down sampled to 250 Hz. No other ECG preprocessing steps were done.

### Model architecture and optimization

The Wake Forest dataset was split into three subsets: a training set (80%), a validation set (10%), and a holdout test set (10%). To prevent overfitting, the data split was performed at the patient level, ensuring that all the ECGs from the same patient resided in the same subset. We modified a convolutional neural network architecture, ResNet [21], by adapting for signal-type data and ECGs. The final model selected was based on the highest area under the receiver operating characteristic curve achieved in the internal validation set. The final model was then implemented in the Wake Forest holdout and PhysioNet datasets (external validation) with no further tuning. Please refer to Supplementary Material SI.1 for details of the model architecture and computational framework.

### Electrocardiographic sex index (ESI) and sex discordance index (SDI)

**Fig. 1:**
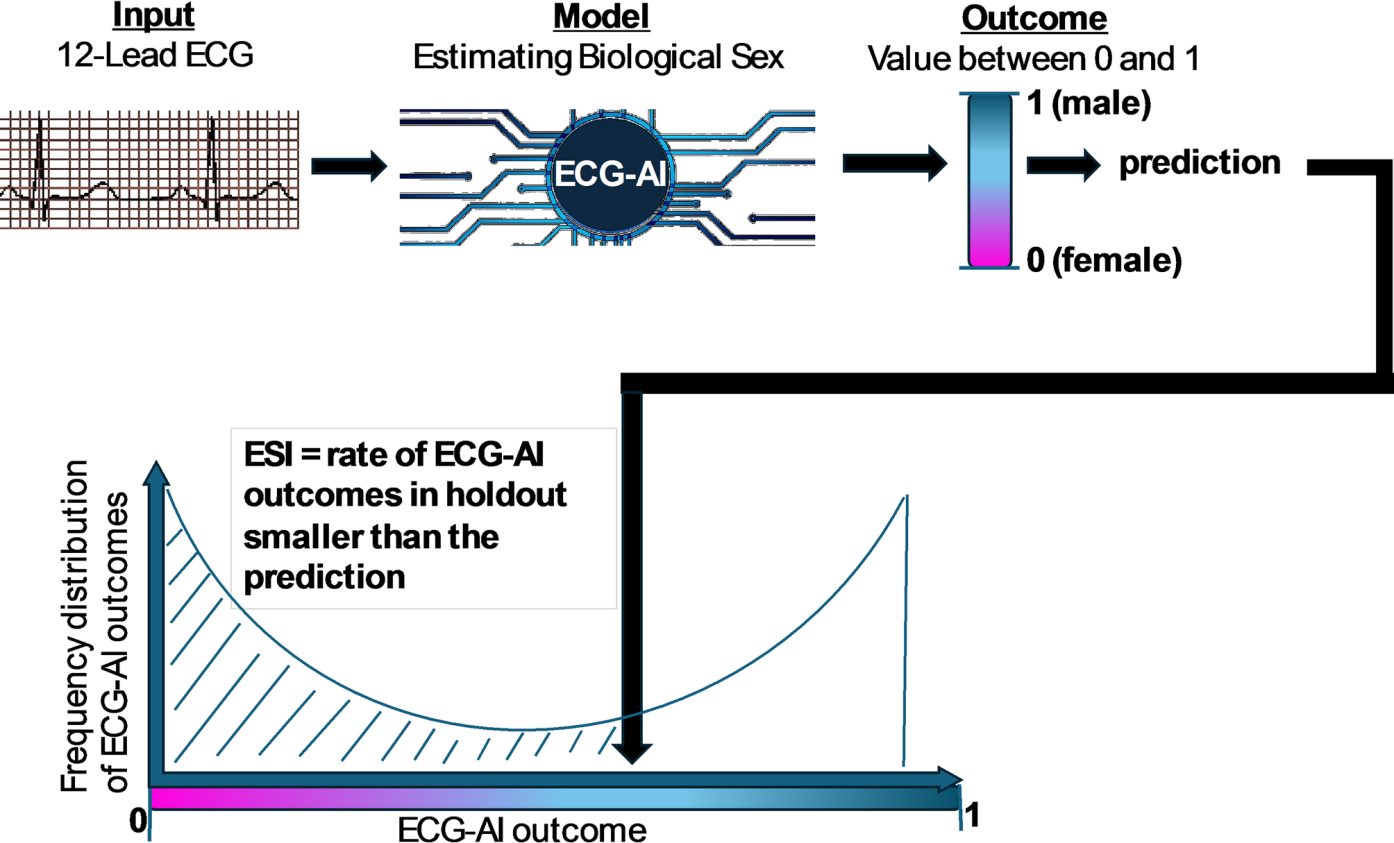
Calculation of the ESI with steps including binary sex detection, ranking of predictions, and generation of ESI

The ECG-AI prediction model outputs range from 0 (most likely female) to 1 (most likely male), with a bimodal distribution. To assess associations with clinical outcomes of interest, the ECG-AI outputs were rank normalized. The ESI was calculated as the proportion of ECG-AI outcomes in the holdout set that were equal to or smaller than the specific ECG-AI output (Fig. 1). A higher ESI indicates that the ECG-AI output is closer to the male end of the spectrum, and a lower ESI suggests a closer alignment with the female end. The ESI for an ECG-AI output $\:{x}_{i}$ is given by:

$$\:ESI\left({x}_{i}\right)=\:\frac{1}{n}\sum\:_{j=1}^{n}I\left({x}_{j}\le\:{x}_{i}\right)$$

where $\:I\left({x}_{j}\le\:{x}_{i}\right)$ is 1 if $\:{x}_{j}\le\:{x}_{i}$ and 0 otherwise, and n is the number of outputs in the holdout set. The SDI represents the difference between binary sex and the ESI. The SDI is calculated as $\:\varvec{S}\varvec{D}\varvec{I}=\varvec{a}\varvec{b}\varvec{s}(\varvec{E}\varvec{S}\varvec{I}-0.5)$.

### Subgroup analyses

We carried out subgroup analyses on the Wake Forest holdout data for race, ethnicity, age, and body mass index (categorized as underweight, normal, overweight, or obese based on World Health Organization (WHO) criteria). The DeLong test [22] was used to compare areas under the curve.

### The role of the ECG sex index in cardiovascular and non-cardiovascular conditions

We then assessed the utility of the ESI in predicting the 1-year risk for all-cause mortality, heart failure, and progression to kidney failure among patients with chronic kidney disease (CKD). Mortality and heart failure are well-established cardiovascular outcomes where sex differences play a crucial role, and kidney failure is strongly influenced by sex-related factors, including eGFR, which is a function of sex.

For each of the three outcomes of interest, we implemented a similar inclusion-exclusion protocol (Supplementary Figure S.1.). ECGs were coded as cases if an event was recorded within one year of the index ECG, or as controls if the event occurred after one year or did not occur at all.

In this retrospective study, Race (1 for Black, 0 otherwise), age (at the time of ECG, in decades, e.g., age 63 as 6.3), and sex (1 for male, 0 for female) were used as predictors in all models for the outcomes of interest, following conventions established in studies like Atherosclerosis Risk in Communities Study [23]. We created five models: Model 1 (binary sex coding), Model 2 (ESI), Model 3 (SDI), Model 4 (binary sex & ESI), and Model 5 (binary sex & SDI). The risk factors differ for all three outcomes, but all represent the patient at the time of index ECG. Please refer to Supplementary Material SI.2 for detailed outcome and risk factor definitions.

- **All-Cause Mortality Risk Prediction.** Data included deaths that occurred in or outside hospitals, using the death indices from North Carolina and surrounding states.
- **Heart Failure Risk Prediction**. The first occurrence of heart failure was determined from International Classification of Disease (ICD) 10 codes and EHR data. In addition to demographic factors, key risk factors for heart failure (heart rate, hypertension, diabetes, valvular disease, and coronary artery disease) from major studies were also included as predictors [24–26]. Heart rate was calculated from the index ECG. All other risk factors were based on ICD-10 codes, whether coded at the time of the index ECG or before.
- **Kidney Failure Risk Prediction**. Kidney failure was defined as the initiation of dialysis or kidney transplant among patients with CKD. Following the methods of Tangri et al. [27]., we included only the estimated glomerular filtration rate in addition to demographic variables to predict the risk for kidney failure. We used the CKD-EPI 2021 equation [28] to estimate the glomerular filtration rate from serum creatinine levels. Only creatinine values from blood drawn within 2 weeks of an ECG recording were used.
- Other specific clinical variables were defined as follows: heart rate was recorded as the ventricular rate from the ECG; hypertension, diabetes, valvular disease, and coronary artery disease were determined using relevant ICD-10 codes.

For each clinical outcome, five different logistic regression models (Models 1–5) were created to obtain odds ratios (ORs) to assess the statistical significance of binary sex, the ESI, and the SDI as independent risk factors. The entire analytical cohort was used for each clinical outcome, and ORs and areas under the curve were calculated.

We also implemented Models 1–5 for all three clinical outcomes via nine different machine learning algorithms, including neural networks (with one, two, or three hidden layers); ensemble models (gradient boosting [29], random forest [30], subspace discriminant [31], subspace KNN [32], and RUS boosted [33]); and logistic regression. Bayesian optimization was used for hyperparameter tuning [34].

For all three clinical outcomes, the same predictive model development and cross-validation strategies were followed. For each clinical outcome and model, once the analytical cohort was identified, the patient data in the cohort were split into 80% derivation and 20% holdout test sets. The derivation cohort was further split into 90% training and 10% validation sets. A fivefold cross-validation machine learning model was built using training data and implemented with the validation data. Among the seven alternative machine learning algorithms, the one with the highest validation area under the curve was selected as the final model. This final model was then applied to the holdout data to report the final predictive performance.

## Results

### ECG-AI model for classifying binary sex from ECGs

The ECG-AI model was trained and validated using ECGs from patients seen at Wake Forest. The final model was tested on the Wake Forest holdout dataset and the PhysioNet data used for external validation. For the Wake Forest holdout dataset, the ECG-AI model yielded an area under the curve of 0.947 (0.946–0.947), with both a sensitivity and specificity of 0.87 in classifying ECGs into clinically recorded male or female categories. The areas under the curve for Caucasian (*n* = 260,282), Black (*n* = 70,443), Asian (*n* = 341), American Indian (*n* = 706), and Hispanic (*n* = 3,696) populations were 0.948 (0.948–0.949), 0.939 (0.937–0.940), 0.939 (0.915–0.963), 0.938 (0.922–0.955), and 0.964 (0.958–0.969), respectively. In the PhysioNet cohort, the ECG-AI model yielded an area under the curve of 0.916 (0.914–0.919). Among Asians, areas under the curve in the external validation cohort and the Wake Forest holdout dataset did not significantly differ (DeLong test, *p* = 0.073).

### Derivation of the ESI

The ESI was calculated for ECGs in the holdout data (*n* = 174,173), as detailed in the Methods section. As expected, the mean (SD) ESI was 0.50 (0.29), whereas it was 0.27 (0.18) for females and 0.72 (0.19) for males (Supplementary Material Figure S.2.).

### Subgroup analyses for the ESI

When racial subgroups were compared, the ESI was lowest in Asian and Hispanic females (Table 1). Among males, the ESI in Caucasians was significantly greater than that in Black and Asian males, but significantly lower than that of Hispanic males. Caucasian and American Indian males had similar ESIs.

**Table 1:** ESI in subgroups

		Female		Male	
		N of ECGs	ESI, Mean (SD)	N of ECGs	ESI, Mean (SD)
All		174,173	0.271 (0.181)	183,126	0.718 (0.185)
Race	**Caucasian**	122,081	0.272 (0.181)	138,516	0.720 (0.182)
	Black	38,243	0.280 (0.183)**	32,284	0.711 (0.192)***
	Asian	341	0.212 (0.180)***	140	0.683 (0.219)*
	American Indian	313	0.251 (0.197)*	394	0.716 (0.198)
	Hispanic	1,792	0.207 (0.173)***	1,915	0.732 (0.198)**
Age	**18–29**	9,341	0.201 (0.176)	8,180	0.761 (0.200)
	30–44	22,893	0.215 (0.173)***	20,662	0.756 (0.193)
	45–59	43,644	0.253 (0.180)***	51,296	0.743 (0.188)***
	60+	98,295	0.298 (0.177)***	102,988	0.695 (0.177)***
BMI	Underweight	2,399	0.334 (0.188)***	1,473	0.645 (0.188)***
	**Normal**	21,517	0.296 (0.184)	22,785	0.716 (0.178)
	Overweight	16,040	0.299 (0.182)	26,570	0.748 (0.177)***
	Obese	18,225	0.294 (0.180)	16,889	0.733 (0.179)***

Bold text indicates the reference category for statistical comparisons.

*indicates significance at ***p < 0.001, **p < 0.01, and *p < 0.05

ESI scores decreased with age in males but increased with age in females. This finding is also supported by the Pearson correlation coefficient between the ESI and age, which was 0.192 (*p* < 0.001) for females and − 0.165 (*p* < 0.001) for males.

When normal weight was used as a reference category, the ESI was significantly lower for underweight females. Underweight males had a significantly lower ESI, and overweight and obese males had a significantly higher ESI compared to men of normal weight.

### Associations among the ESI, clinical outcomes, and the SDI

**Fig. 2:**
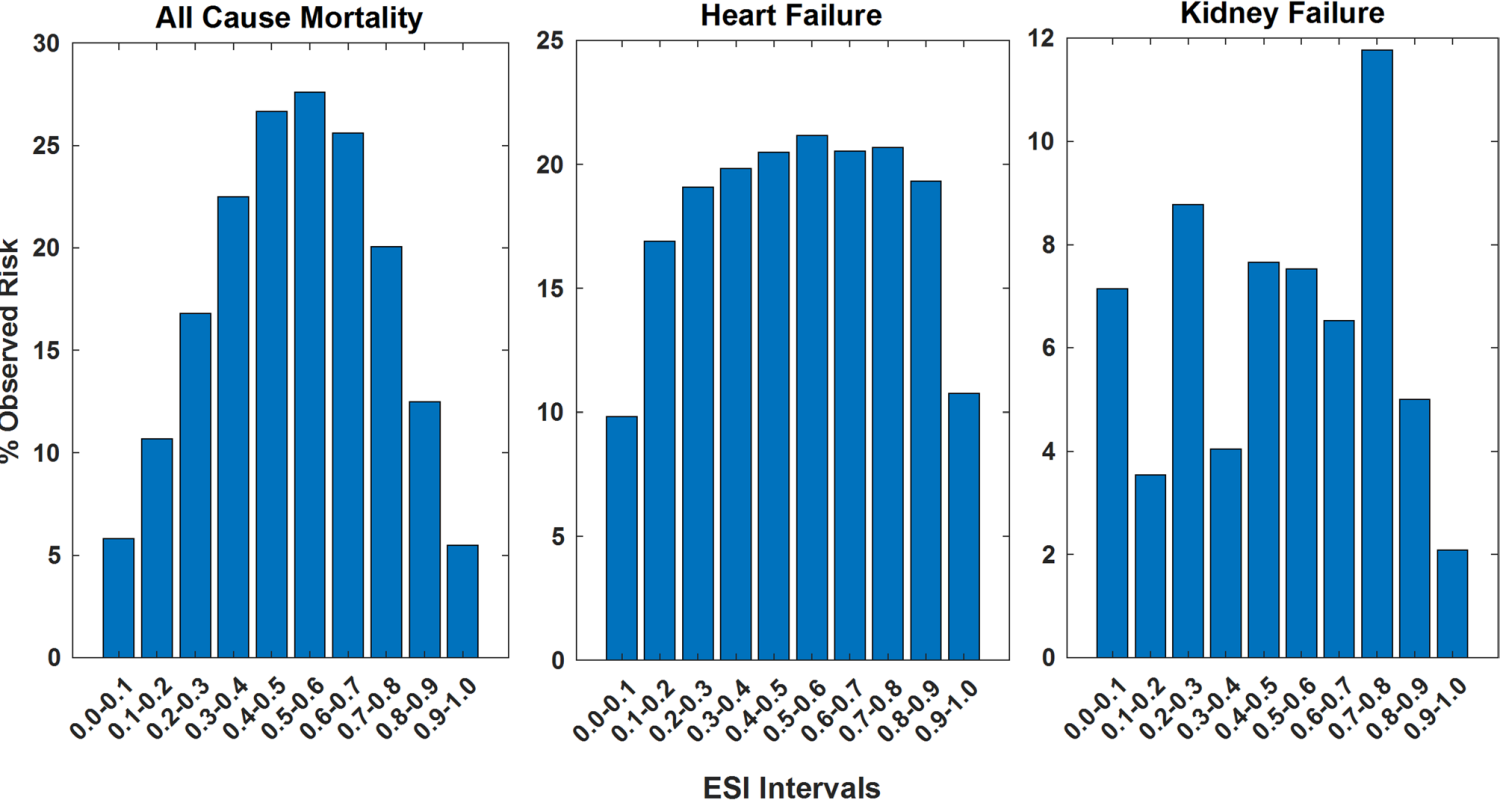
Observed prevalence of mortality (LEFT), Heart Failure (Middle) and Kidney Failure (Right) at different ESI intervals

We plotted the observed incidence rates at different ESI intervals for the three clinical outcomes of interest in Fig. 2.

Figure 2 shows that the direction of the effects of ESI values far from most likely female (0) and most likely male (1) are associated with significantly higher rates of mortality and higher rates of heart failure while the pattern differed for the 1-year risk for kidney disease, reflecting the significantly smaller cohort (*n* = 1,236) and event rate (6.7%) compared with the 1-year risk for all-cause mortality (*n* = 101,339, mortality rate of 17.6%) and heart failure (*n* = 74,846, event rate of 13.6%).

### Role of the ESI in predicting the 1-year risk for the three clinical outcomes

The characteristics of the analytical cohorts for all three clinical outcomes are presented in Table 2.

**Table 2:** Cohort characteristics for each clinical outcome

Prediction of 1-year risk for:	Model Variables	*n* (%) or mean (SD)
All-cause mortality (*n* = 101,339)	Mortality Within 1 year	17,788 (17.6)
	Male Sex	52,282 (51.6)
	ESI	0.49 (0.29)
	Age	62.08 (16.29)
	Black Race	20,313 (20.0)
Heart failure (*n* = 74,846)	Heart Failure Within 1 year	10,201 (13.6)
	Male Sex	36,915 (49.3)
	ESI	0.48 (0.41)
	Age	60.51 (15.70)
	Black Race	16,990 (22.7)
	Heart Rate	79.46 (21.11)
	Hypertension	35,169 (47.0)
	Diabetes	16,991 (22.7)
	Valvular Disease	8,001 (10.7)
	Coronary Artery Disease	14,612 (19.5)
Kidney failure (*n* = 1,236)	Kidney Failure Within 1 year	89 (6.7)
	Male Sex	637 (48.1)
	ESI	0.48 (0.23)
	Age	70.40 (14.68)
	Black Race	391 (29.5)
	eGFR	48.45 (29.67)

The logistic regression models for each clinical outcome for the entire analytical cohort are reported in Table 3. Table 3 also includes the hold-out areas under the curve obtained from the best machine learning model.

**Table 3:** Logistic regression models in the overall analytical cohort

Clinical Outcome, total	Input Variables	Odds Ratios				
		M1: Binary Sex	M2: ESI	M3: SDI	M4: Sex & ESI	M5: Sex & SDI
All-cause mortality (*n* = 101,339, ACM rate = 17.6%)	Sex	1.24 (1.22–1.31)***			1.40 (1.32–1.49)***	1.40 (1.32–1.49)***
	ESI		1.24 (1.16–1.33)***		0.78 (0.70–0.87)***	
	SDI			0.80 (0.75–0.86)***		1.28 (1.16–1.43)***
	Age (10y)	1.62(1.50–1.54)***	1.52 (1.50–1.54)***	1.52 (1.50–1.54)***	1.52 (1.50–1.54)***	1.50 (1.50–1.54)***
	Race	1.13 (1.08–1.19)***	1.12 (1.06–1.17)***	1.12 (1.06, 1.17)***	1.13 (1.07–1.18)***	1.136 (1.07–1.18)***
AUC LR using all data		0.67 (0.66–0.67)	0.67 (0.66–0.67)	0.67 (0.66 67)	0.67 (0.67–0.68)	0.67 (0.67 0.68)
AUC of ML on holdout data		0.67 (0.66–0.68)	0.72 (0.71–0.73)***	0.71 (0.70–0.71)***	0.72 (0.72–0.73)	0.71 (0.71–0.72)
Heart failure (*n* = 74,846, event rate = 13.6%)	Sex	1.04 (0.99–1.09)			1.05 (0.98–1.14)	1.01 (0.96–1.05)
	ESI		1.03 (0.97–1.09)		0.98 (0.89–1.08)	
	SDI			0.23 (0.19–0.26)***		0.23 (0.20–0.27)***
	Age (10y)	1.42 (1.40–1.45)***	1.43 (1.41–1.46) ***	1.40 (1.37–1.42)***	1.42 (1.40–1.45) ***	1.40 (1.38–1.42) ***
	Race	1.27 (1.20–1.35) ***	1.141 (1.07–1.220) ***	1.26 (1.19–1.33)***	1.29 (1.21–1.35) ***	1.29 (1.19–1.33) ***
	Heart Rate	1.02 (1.02–1.02) ***	1.02 (1.02–1.02) ***	1.01 (1.01–1.02)***	1.02 (1.02–1.02) ***	1.01 (1.01–1.02) ***
	Hypertension	0.84 (0.80–0.89) ***	0.84 (0.80–0.89) ***	0.85 (0.80–0.89)	0.84 (0.80–0.89) ***	0.85 (0.80–0.90) ***
	Diabetes	1.99 (1.89–2.09) ***	1.99 (1.89–2.09) ***	1.94 (1.85–2.05)***	1.99 (1.89–2.09) ***	1.94 (1.85–2.05) ***
	Valvular Disease	2.10 (1.98–2.23) ***	2.11 (1.99–2.24) ***	2.08 (1.96–2.21)***	2.10 (1.98–2.23) ***	2.08 (1.96–2.21) ***
	Coronary Artery Disease	2.76 (2.62–2.91) ***	2.77 (2.63–2.92) ***	2.76 (2.62–2.90)***	2.76 (2.62–2.91) ***	2.76 (2.62–2.90) ***
AUC LR using all data		0.76 (0.76–0.77)	0.76 (0.76–0.77)	0.77 (0.77–0.77)	0.76 (0.76–0.77)	0.77 (0.77–0.78)
AUC of ML on holdout data		0.78 (0.77–0.79)	0.79 (0.78–0.80)	0.81 (0.80–0.82)***	0.79 (0.78–0.80)	0.85 (0.85–0.86)***
Kidney failure (*n* = 1,236, event rate = 6.7%)	Sex	2.58 (1.50–4.51)***			3.07 (1.53–6.16)*	2.62 (1.46–4.61)*
	ESI		2.33 (0.73–7.45)		0.54 (0.12–2.42)	
	SDI			0.82 (0.10–6.59)		1.46 (1.18–12.27)
	Age (10y)	0.57 (0.47–0.66)***	0.55 (0.47–0.65)***	0.56 (0.48–0.66)***	0.56 (0.47–0.67)***	0.56 (0.47–0.66)***
	Race	1.24 (0.70–2.18)	0.95 (0.55–1.63)	0.86 (0.51–1.46)	1.22 (0.69–2.16)	1.22 (0.69–2.17)
	eGFR	0.91 (0.89–0.93)***	0.91 (0.89–0.93)***	0.91 (0.89–0.93)***	0.91 (0.89–0.93)***	0.91 (0.89–0.93)***
AUC LR using all data		0.89 (0.87–0.92)	0.89 (0.86–0.93)	0.89 (0.86–0.93)	0.90 (0.86–0.93)	0.90 (0.86–0.93)
AUC of ML on hold out data		0.83 (0.75–0.90)	0.92 (0.92–0.98)	0.96 (0.94–0.99)*	0.89 (0.84–0.95)	0.98 (0.96–0.99)**

For odds ratios: * significant at *p* < 0.05, ** significant at *p* < 0.01, *** significant at *p* < 0.001

For areas under the curve: * significant at *p* < 0.05, ** significant at *p* < 0.01, *** significant at *p* < 0.001 compared to Model 1 using DeLong test

AUC = areas under the curve; eGFR = estimated glomerular filtration rate; LR = logistic regression; ML = machine learning

Despite methodological differences, the OR for binary sex in our M1 benchmark models for all-cause mortality, heart failure, and kidney failure aligns with prior studies [35–37]. For all three outcomes, all five logistic regression models had similar areas under the curve. For all-cause mortality, all three methods of sex representation yielded statistically significant ORs in Models 1, 2, and 3. For heart failure, only the SDI yielded a statistically significant OR, and for kidney failure, only binary sex yielded a statistically significant OR.

For all-cause mortality, the ESI and the SDI (Models 2 and 3, respectively) yielded significantly greater areas under the curve than binary sex (Model 1). The use of the ESI or SDI together with binary sex did not further improve that result. For heart failure, both the ESI and the SDI yielded better areas under the curve than binary sex, but the increase was significant only for the SDI (Model 3 versus Model 1). However, binary sex together with the SDI further significantly improved the area under the curve compared with binary sex alone (Model 5 versus Model 1). For kidney failure, both the ESI and the SDI and their joint use with binary sex improved the area under the curve compared binary sex alone. However, considering the smaller sample and event rates for kidney failure, the increases were not significant for Models 2 and 4, although they were for Models 3 and 5 when the SDI was used.

## Discussion

Some sex-related differences in the human cardiovascular system already exist at birth and are primarily due to purely biological mechanisms, such as genes and sex hormones [2]. Sex-related differences in cardiovascular risk and the varying associations of traditional cardiometabolic risk factors have been intensive areas of research [38]. For instance, males typically have higher mortality rates than females [35], while females have a higher incidence of CKD but are less likely to progress to kidney failure compared to males [36]. Additionally, females are more likely to have heart failure with preserved ejection fraction (HFpEF), while males are more prone to have heart failure with reduced ejection fraction (HFrEF) [37]. However, to date, sex has been treated as a binary risk factor when assessing its association with clinical outcomes.

The role of sex in ECGs is well known [4, 39, 40] and has been described as an independent risk factor for different types of arrhythmias [4, 41–44]. Sex hormone levels may play a direct role in ECG changes [39, 44, 45]. For example, Attia et al.. trained a convolutional neural network model using 12-lead, 10-second ECG signals to classify sex and obtained an area under the curve of 0.97 [46], suggesting significant differences in the electric activity of the heart in men and women. Indeed, ECGs may reflect the multifactorial influences that drive sex, such as sex hormones. To support this idea, Naser et al.. analyzed the relationship between the sex probability derived from an ECG-AI algorithm and sex hormone levels [47]. In that study, total testosterone levels were lower, and estradiol levels were higher with decreasing ECG-AI male probability in both sexes. Male and female patients with discordant ECG-AI sex probabilities had significantly different testosterone or estradiol levels, respectively.

Despite this literature, no study has taken the next step in redefining sex in risk models. In this study, we propose a novel continuous representation of sex, the ESI, as a risk factor for both cardiovascular and non-cardiovascular risk, where sex is hypothesized to be associated with outcomes. We based the derivation of the ESI on the rank normalization of the outcomes of a convolutional neural network model that classifies ECGs into males and females. This model achieved generalizable results by providing a holdout area under the curve of 0.95 and an external validation area under the curve of 0.92. The high accuracy of the external validation data– an arrhythmia dataset from two hospitals in China, with distinctly different patients compared to the derivation cohort– suggests the generalizability of the convolutional neural network model for detecting sex.

Next, the ESI was calculated for a given ECG, providing a value between 0 and 1. The ESIs of Caucasian and Black females were similar, but they were lower for Asian, American Indian, and Hispanic females. Among males, Hispanic males had the highest ESI, followed by Caucasians and American Indian males with similar ESIs, which was lower for black males and lowest for Asian males. The ESI significantly increased with age in females but decreased in males.

We also showed that the associations between the ESI and clinical outcomes were not linear, where the risk is greater when the ESI is far from 0 to 1. To account for this association, we also created the SDI and assessed its utility.

Our results for all three clinical outcomes show that the ESI and SDI are similar to binary sex in terms of prediction accuracy. The SDI yielded a statistically significant OR in models for kidney failure, unlike binary sex and heart failure. However, the ESI and SDI provide higher areas under the curve when used in machine learning models. When the SDI was used together with binary sex, it further improved the area under the curve for heart failure and kidney failure outcomes. These results suggest that the ESI and its derivatives can represent binary sex on a continuous scale and perform as well as binary sex in linearizable models such as logistic regression but offer improved predictive performance in machine learning models. In clinical practice, these indices could refine risk stratification, particularly for conditions where sex differences are relevant, such as heart failure and kidney disease. Integrating ESI and SDI into predictive models may improve early identification of at-risk individuals, ultimately aiding in more personalized clinical decision-making.

### Limitations

There are several limitations to our study. First, ECGs were recorded as a part of the standard of care. Therefore, there was selection bias toward people whose health condition required an ECG. Second, we used sex information entered into ECG devices during ECG recordings, which may include some level of data entry error. Furthermore, the information may not represent sex at birth or the sex with which individuals identify. Additionally, karyotype data were unavailable, meaning individuals with atypical chromosomal patterns or nonbinary identities could not be identified, potentially influencing ECG-based sex index distributions. Finally, many other medical conditions are associated with sex. Future studies are needed to assess the role of the ESI in any risk calculator compared with a binary sex variable.

## Conclusions

Sex is a complex biological, physiologic, and genetic phenomenon that cannot be expressed with a simple binary variable, as has been used in the literature. The ESI, with a continuous representation of sex, shows potential as a more accurate predictor of both cardiovascular and non-cardiovascular outcomes. The SDI also emerged as a significant predictor of all-cause mortality, heart failure, and kidney failure. Adding the ESI and/or SDI should be considered in predictive models of outcomes where sex is hypothesized to be a risk factor.

## Electronic supplementary material

Below is the link to the electronic supplementary material.

Supplementary Material 1

## Data Availability

The study use two main data sources (1) EHR data from Wake Forest Baptist Health and (2) public data from PhysioNet. EHR data is not available to share due to institutional policies. The public data is accessible for anyone through PhysioNet. Following publication, the code for calculation of ESI will be shared with public without any need for a request through GitHub (https://github.com/akbilgic/ESI/).
