# Supplementary material for: Electrocardiographic sex index: a continuous representation of sex
Source: Biol Sex Differ. 2025 Jul 17;16:53. doi: 10.1186/s13293-025-00727-2 (PMC12273486; doi:10.1186/s13293-025-00727-2)
Supplement: Supplementary file 1 — Supplementary Material 1 [file 13293_2025_727_MOESM1_ESM.docx]

**Electrocardiographic Sex Index: A Continuous Representation of Sex**

**SUPPLEMENTARY MATERIAL**

**Table of Contents 1**

**SI.1 ECG-AI Model Architecture and Computational Framework 2**

**Figure S.1. Case and Control Determination 3**

**SI.2. Risk Factor and Clinical Outcome Definitions 4**

**Figure S.2. The Frequency Distribution of ESI by Sex 6**

**Supplementary Document References 7**

**SI.1. ECG-AI Model Architecture and Computational Framework**

We employed a modified ResNet [1] architecture adapted for signal type data in ECGs. The output layer consisted of two neurons, representing the binary classification of sex. A categorical cross-entropy loss function was employed, along with a dense layer activated by the softmax function as the penultimate layer. The Adam optimizer [2] was used to optimize the weights of the model, with default hyperparameters (learning_rate = 0.001, beta_1 = 0.9, beta_2 = 0.999), and a clipnorm of 1 as a regularizer. Additionally, a learning rate reduction strategy was employed, halving the learning rate if there was no improvement in validation loss for 15 consecutive epochs, with a minimum learning rate of 0.0001. The training process with a maximum epoch of 100 was stopped if there was no improvement in validation area under the receiver operating characteristics curve for 30 consecutive epochs. The final model selected was based on the highest area under the curve achieved in the internal validation dataset. The final model was then implemented on the holdout and external validation datasets without any further tuning.

Model training was conducted with a batch size of 512 utilizing multiple GPUs (Tesla V100 x 3) for parallel computing, optimizing computational efficiency and reducing training time. The TensorFlow and Keras libraries in a Python environment were used to build sex classifications. Matlab 2023a was used for ESI calculation and machine learning model building. SPSS 29.0.2.0 was used for descriptive statistics and logistic regression analyses.

**Figure S.1. Case and Control Determination 5**

**SI.2. Clinical outcome definitions**

- **All-cause mortality**. All-cause mortality data were retrieved from the Wake Forest EHR which includes both deaths that occurred in-hospital and data from the North Carolina Death Index. Because some patients at Wake Forest come from nearby states, we also gathered information from the Virginia, West Virginia, Tennessee, South Carolina, and Florida Death Indices.
- **Heart Failure.** Heart failure occurrence and its date were determined using ICD-10 codes and their first occurrence documented in the EHR. The diagnostic codes for heart failure included left ventricular failure (I50.1), systolic heart failure (I50.2), diastolic heart failure (I50.3), combined systolic and diastolic heart failure (I50.4), other heart failure (I50.8), or unspecified heart failure (I50.9). The subclassifications of these main categories were also considered [3].
  - **Heart Rate**. Heart rate variable was used as ventricular rate calculated by ECG equipment for each index ECG.
  - **Hypertension.** Hypertension was determined based on ICD-10 codes for primary hypertension (I10), hypertensive heart disease without heart failure (I119), renovascular hypertension (I150), hypertension secondary to other renal disease (I151), hypertension secondary to endocrine disorders (I151), other secondary hypertension (I158), unspecified secondary hypertension (I159), hypertensive urgency (I160), hypertensive emergency (I161), or unspecified hypertensive crisis (I169).
  - **Diabetes.** Diabetes was determined based on ICD-10 codes for diabetes mellitus due to underlying condition (E08), diabetes mellitus due to underlying condition (E09), Drug or chemical induced diabetes mellitus (E10), Type 1 diabetes mellitus (E11), Type 2 diabetes mellitus (E12), or other specified diabetes mellitus (E13).
  - **Valvular Disease.** Valvular disease was determined based on ICD-10 codes for rheumatic mitral valve diseases (I05), rheumatic aortic valve diseases (I06), rheumatic tricuspid valve diseases (I07), multiple valve diseases (I08), and their subclassifications.
  - **Coronary Artery Disease.** Coronary artery disease was determined based on ICD-10 codes for atherosclerotic heart disease of native coronary artery without angina pectoris (I25.10), atherosclerotic heart disease of a native coronary artery with unstable angina pectoris (I25.110), ischemic cardiomyopathy (I25.5), silent myocardial ischemia (I25.6), atherosclerosis of coronary artery bypass graft(s) and coronary artery of transplanted heart without angina pectoris (I25.700), atherosclerosis of coronary artery bypass graft(s) and coronary artery of transplanted heart with unstable angina pectoris (I25.710), atherosclerosis of coronary artery bypass graft(s) and coronary artery of transplanted heart with angina pectoris (I25.810), chronic ischemic heart disease, unspecified (I25.9), or other forms of chronic ischemic heart disease (I25.89).
- **Kidney Failure among patients with chronic kidney disease (CKD).** CKD was identified using ICD-10 codes N18.X, and kidney failure was defined by Current Procedural Terminology (CPT) codes for initiation of dialysis (CPTs 90945, 90947, 90935, 90937, 90935, 90937, 90960, 90961, 90962, 90963, 90964, 90965, 90966) or kidney transplant (CPT 5569).
  - Estimated Glomerular Filtration Rate. We used the CKD-EPI 2021 creatinine [4] formula for estimation of glomerular filtration rate as given below:

$eGFR=142 {min(\frac{Scr}{x},1)}^{a_{1}}{max(\frac{Scr}{x},1)}^{-1.2}{0.9938}^{Age}d$ where Scr is serum creatinine, x is 0.7 for females and 0.9 for males, a1 is -0.241 for females and -0.302 for males, and d is 1.012 for females and 1 for males.

**Figure S.2. The Frequency Distribution of ESI by sex**

**Supplementary Document References**

[1] K. He, X. Zhang, S. Ren, and J. Sun, "Deep residual learning for image recognition," in *Proceedings of the IEEE conference on computer vision and pattern recognition*, 2016, pp. 770-778.

[2] D. Kingma and J. Ba, "Adam: A Method for Stochastic Optmization," *arXiv,* vol. arXiv:1412.6980, 2014.

[3] B. A. Bates *et al.*, "Validity of International Classification of Diseases (ICD)-10 Diagnosis Codes for Identification of Acute Heart Failure Hospitalization and Heart Failure with Reduced Versus Preserved Ejection Fraction in a National Medicare Sample," *Circulation: Cardiovascular Quality and Outcomes,* vol. 16, no. 2, p. e009078, 2023/02/01 2023, doi: 10.1161/CIRCOUTCOMES.122.009078.

[4] A. Inker Lesley *et al.*, "New Creatinine- and Cystatin C–Based Equations to Estimate GFR without Race," *New England Journal of Medicine,* vol. 385, no. 19, pp. 1737-1749, 2021/11/03 2021, doi: 10.1056/NEJMoa2102953.
